# Predictors of Minimal Hepatic Encephalopathy in Patients with Cirrhosis

**DOI:** 10.4103/1319-3767.65189

**Published:** 2010-07

**Authors:** Praveen Sharma, Barjesh C. Sharma

**Affiliations:** Department of Gastroenterology, G. B. Pant Hospital, New Delhi, India

**Keywords:** Cirrhosis, minimal hepatic encephalopathy, predictors

## Abstract

**Background/Aim:**

Minimal hepatic encephalopathy (MHE) impairs patient’s daily functioning of life. Predictors of MHE in cirrhotic patients have not been evaluated.

**Patients and Methods:**

A total of 200 cirrhotic patients (Child A, 74 [37%]; Child B, 72 [36%]; Child C, 54 [27%]) were evaluated by psychometry, P300 auditory event-related potential (P300ERP) and critical flicker frequency (CFF). MHE was diagnosed by abnormal psychometry (>2 S.D.) and P300ERP (>2.5 S.D.). Univariate and multivariate logistic regression analyses were performed to determine the predictors of MHE.

**Results:**

Eighty-two (41%) patients were diagnosed to have MHE – 26/74 (35%) in Child A, 26/72 (36%) in Child B and 30/54 (56%) in Child C. Ninety-seven (48.5%) patients had abnormal psychometric tests, and 96 (48%) had prolonged P300ERP (>358 ms). Sixteen (16.5%) patients with abnormal psychometry had P300ERP < 358 ms, and 15 (14.5%) patients with normal psychometry results had P300ERP > 358 ms. One hundred and three patients had CFF value < 39 Hz with specificity of 86.6% and sensitivity of 72.9% for MHE. Model for end-stage liver disease (MELD) (17.9 ± 5.7 vs. 13.4 ± 4.2, *P* = 0.005), Child-Turcotte-Pugh (CTP) score (8.4 ± 2.5 vs. 7.7 ± 2.2, *P* = 0.02), ammonia (104.8 ± 37.9 vs. 72.5 ± 45.2 µmol/L, *P* = 0.001) and CFF (37.0 ± 2.8 vs. 41.0 ± 3.4 Hz, *P* = 0.001) were significantly higher in MHE as compared to non-MHE patients. Ninety-one (45.5%) patients had MELD > 15.5, 115 (57.5%) had CTP score > 7.5, while 93 (46.5%) had venous ammonia > 84.5 µmol/L. On univariate analysis, MELD (8.52 [95% CI, 4.46-16.26; *P* = 0.001]), CFF (17.34 [95% CI, 8.16-36.85; *P* = 0.001]) and venous ammonia (7.80 [95% CI, 4.11-14.81; *P* = 0.003]) were associated with MHE; while CTP score (1.51 [95% CI, 0.85-2.69; *P* = 0.30]) was not significant. On multivariate analysis, MELD, CFF and venous ammonia were predictive of MHE.

**Conclusion:**

Prevalence of MHE in this study was 41%; and MELD > 15.5, CFF < 39 Hz and venous ammonia > 84.5 µmol/L were predictive of MHE.

Minimal hepatic encephalopathy (MHE) is a crucial disorder that may seriously impair patient’s daily functioning and quality of life.[1–3] The prevalence of MHE varies between 30% and 55% in patients with liver cirrhosis, dependent on the diagnostic criteria used.[3–9] Some authors proposed testing all cirrhotics to identify patients with MHE, based on several studies that have shown improvement of MHE with therapy.[6,7–14] It has been shown that therapy may improve quality of life or delay the development of an episode of hepatic encephalopathy (HE).[12–15] However, few hepatologists really screen patients for MHE due to time-consuming neuropsychological and neurophysiological tests.[15,16] Hence there is need for knowing predictors of MHE in cirrhotic patients. Model for end-stage liver disease (MELD) and Child-Turcotte-Pugh (CTP) scores were recognized as predictors of long-term survival and complications in patients with cirrhosis.[17–21] Controversy exists in literature regarding the correlation between severity of liver disease as assessed by CTP and MELD scores and prevalence of MHE.[7–9,22–24] Gut-derived nitrogenous substances are universally acknowledged to play a major role in the pathogenesis of HE, and pathogenesis of MHE is thought to be similar to that of overt HE. Specifically, ammonia is thought to be a critical factor in the pathogenesis.[25,26] Recently, critical flicker frequency (CFF) has been used for the diagnosis of MHE, as many of the problems encountered with doing psychometric tests can be circumvented.[27–29] However, factors associated with MHE have not been fully evaluated. In this study, we analyzed predictors associated with MHE in patients with cirrhosis.

## PATIENTS AND METHODS

### Patient population

Two hundred ninety consecutive cirrhotic patients without HE were screened prospectively for MHE in a single center. Cirrhosis was diagnosed on a clinical basis involving laboratory tests, endoscopic evidence, sonographic findings and liver histology if available. Following were the etiologies of cirrhosis: alcohol (*n*= 98), chronic hepatitis B (*n*= 80), chronic hepatitis C (*n*= 64), autoimmune hepatitis (*n*= 10), primary biliary cirrhosis (*n*= 4) and cryptogenic cirrhosis (*n*= 34). The exclusion criteria were the presence of overt HE or history of HE; history of taking lactulose or any antibiotics, alcohol intake, gastrointestinal hemorrhage or spontaneous bacterial peritonitis during the past 6 weeks, previous TIPS or shunt surgery, significant comorbid illness such as heart, respiratory or renal failure; and history of any neurologic disease such as Alzheimer’s disease, Parkinson’s disease or nonhepatic metabolic encephalopathies. Patients on psychoactive drugs, such as antidepressants or sedatives, were excluded. History of alcohol intake was assessed by either asking the patient or his/ her relative. All patients who were enrolled in the study were evaluated using psychometric tests, P300 auditory event–related potential (P300ERP) and CFF on the same day. Informed consent was taken and the protocol was approved by the hospital ethical committee in accordance with the ethical guidelines of the 1975 Declaration of Helsinki.

### Psychometric testing

All patients underwent number connection tests (NCT-A, NCT-B); and figure connection tests (FCT-A, FCT-B) if illiterate. These tests were done by the authors of this study. Tests were considered abnormal when test score was more than mean +2 standard deviations in comparison with that of age- and education-matched controls.[8] In the NCT-A, which measures cognitive motor abilities, patients connect numbers from 1 to 25 printed on paper as quickly as possible. In the NCT B, letters are also included and the patients connect alternating numbers and letters (1-A-2-B-3 … L-13). In principle, the FCT is similar to the NCT, except that numbers are replaced by figures. Each circle has one to five motifs, thus giving the required 25 figures. In FCT-A, all circles with same motif were connected in order of increasing numbers of motifs and in sequences specified in the chart; while in FCT-B, all circles with one motif were connected in the sequence specified in the chart. Psychometric test was considered abnormal when both NCT-A and NCT-B or FCT-A and FCT-B were abnormal. 161 patients did NCT (A, B), while 39 patients could do only FCT (A, B) as they were illiterate.

### P300 auditory event–related potential

The event-related P300 wave is the most consistent wave and can be considered the electrophysiological counterpart of the psychometric tests as both involve active use of cognitive faculties. P300ERP response was elicited by the standard ‘auditory odd ball paradigm.’ The first major positive peak 250-500 ms for the rare tone was identified as the P300 response and was marked. Latency was measured from the point of stimulus to the peak of P300 waveform in milliseconds. Fifty healthy controls without evidence of acute or chronic liver disease served as age-matched controls for P300ERP. P300ERP latency was considered abnormal if it was above +2.5 standard deviations when compared with the mean latency measured in age-matched controls.

### Measurement of critical flicker frequency threshold

Critical flicker frequency is a newer diagnostic test, a bedside tool for the diagnosis of MHE, and has shown higher sensitivity and specificity compared to psychometric tests for the diagnosis of MHE.[27–29] CFF threshold measures visual discrimination and general arousal. CFF was done by HEPAtonorm analyzer (Accelab GmbH, D-72127 Kusterdingen, Germany) at bedside. Patients were first instructed and trained about the procedure. Flicker frequencies were measured eight times and the mean value was calculated. Measurement of the CFF thresholds was done by intrafoveal stimulation with a luminous diode. Decreasing the frequency of the light pulses from 60 Hz downward, the CFF threshold was determined as the frequency when the impression of fused light turned to a flickering one. CFF was considered abnormal when it was less than 39 Hz.[27]

### Assessment of minimal hepatic encephalopathy

Minimal hepatic encephalopathy was defined by abnormal psychometric study (NCT-A and NCT-B or FCT-A and FCT-B) and abnormal P300 auditory event–related potential. Patients with only abnormal psychometric test or only abnormal P300ERP were not considered to have MHE.

### Blood tests and biochemical examinations

After overnight fasting, patient venous blood was taken and analyzed for routine liver function tests and hematologic parameters by conventional methods. Venous ammonia concentration was determined immediately after the psychometric testing. Venous ammonia was measured within three minutes of blood sampling by using the ammonia checker II (Daiichi Kagaku Co. Ltd., Kyoto, Japan). Ultrasound abdomen and Doppler were done to evaluate for large spontaneous shunts.

### Calculation of MELD and CTP score

Both the scores were determined on the day of evaluation of MHE. CTP score was obtained according to classification proposed by Pugh.[30] The MELD score was calculated according to the following formula: MELD = (0.957 × Log [creatinine in mg/dL] + 0.378 × Log [bilirubin in mg/dL] + 1.12 × Log International normalized ratio [INR] + 0.643) × 10. Minimal values are set to 1.0 for calculation purposes. The maximal serum creatinine level considered within the MELD score equation is 4.0 mg/dL.[31]

### Statistical analysis

Data were expressed as mean ± S.D. For a comparison of categorical variables, chi-square test and Fisher exact test were used; and for continuous variables, a Mann-Whitney *U* test for unpaired data and a Wilcoxon rank sum test for paired data were used as appropriate. Receiver operating characteristic (ROC) analysis was used to identify the threshold values. Area under the curve (AUC) and sensitivity and specificity for cutoff points obtained were reported. Correlations between different tests were calculated by using Spearman–Rho rank correlation. A logistic regression model was used to calculate univariate and multivariate odds ratios (ORs), and their 95% confidence intervals (CIs) were used to identify factors associated with MHE. A significance level of 0.05 was used in all analyses. The statistical analysis was done using SPSS version 10.0 software (SPSS, Chicago, IL).

## RESULTS

200 (age, 41.6 ± 11.7 years; M:F ratio, 159:41) were included, and 90 patients were excluded due to recent upper gastrointestinal bleed,[25] hepatic encephalopathy (*n*= 42), mature cataract (*n*= 4), hepatocellular carcinoma (*n*= 8) and history of antibiotic intake due to various reasons in the past one month (*n*= 11). 74 (37%) patients were in Child A grade, while 72 (36%) and 54 (27%) patients were in Child B and C grades, respectively [Table 1]. 82 (41%) patients were diagnosed as having MHE. Of all the patients, 26/74 (35%) were in Child A grade; 26/72 (36%), in Child B grade; and 30/54 (56%), in Child C grade. There was significant difference in Child C *vs*. Child A and B grades (*P* = 0.03), while there was no difference between Child A and Child B grades. Large spontaneous shunt was seen in 32 patients (19 in MHE group and 13 in non-MHE group, *P* = 0.26).

**Table 1:** Demographic, clinical and biochemical characteristics of the study group

	Cirrhotic (mean±S.D.)
*n*	200
Age (years)	41.6 ± 11.7
ALT (U/L)	50.4 ± 26.0
AST (U/L)	57.6 ± 33.0
Bilirubin (mg/dL)	1.9 ± 1.1
Creatinine (mg/dL)	1.0 ± 0.3
INR	2.09 ± 0.7
Child A	74 (37)
Child B	72 (36)
Child C	54 (27)

Figures in parentheses are in percentage

### Results of psychometric tests

Out of 200 patients, 39 patients were illiterate (those unable to read and write), 124 patients were undergraduates (≤12 years of formal education) and 37 patients were graduates (holders of bachelor's degree with 15 years of formal education). Of the 200 patients, 161 (80.5%) patients could do NCT, while FCT was done by 39 (19.5%) patients as they were illiterate. 97 (48.5%) patients had abnormal psychometric test results [Table 2].

**Table 2:** Prevalence of abnormal results of psychometric tests in 200 cirrhotic patients

Psychometric tests	*n*	*n* (%) Abnormal
Number connection test A	161	74 (46)
Number connection test B	161	82 (51)
Figure connection test A	39	23 (59)
Figure connection test B	39	23 (59)

### Determination of P300 auditory event–related potential in controls and in cirrhotics

P300ERP in controls (*n*=50) was 326.8 ± 12.5 (range, 298-351) ms. P300ERP was considered abnormal when it was >2.5 S.D. of value in controls (358 ms). Of the 200 patients, 96 (48%) had prolonged P300ERP. Patients with abnormal psychometry results had prolonged P300ERP (385.3 ± 27.6 ms) in comparison with those with normal psychometry results (328.9 ± 24.3 ms; *P* < 0.005). Only 16 (16.5%) patients with abnormal psychometry results had P300ERP less than 358 ms, and 15 (14.5%) patients with normal psychometry results had P300ERP greater than 358 ms.

### Critical flicker frequency in controls and in cirrhotics

CFF in controls (*n*= 50) was 41.8 ± 1.9 Hz. Patients diagnosed as MHE had significantly lower values as compared to non-MHE patients (37.0 ± 2.8 *vs*. 41.1 ± 3.4 Hz; *P* = 0.001). At cutoff of 39 Hz, there were 103 patients with CFF value < 39Hz with specificity of 86.6% and the sensitivity of 72.9% for MHE. When taking cutoff at 38 Hz (28) the specificity was 56.4% and sensitivity was 84.7%. Patients who had CFF >39 versus those with CFF< 39 had significant difference in psychometric tests [NCT-A] (29.1±12.3 *vs*. 50.4±14.0 sec, *P* = 0.001) NCT-B (62.2±40.7 *vs*. 133.9±42.9 sec, *P* = 0.001), FCT-A (38.1±25.7 *vs*. 64.4±19.8 sec, *P* = 0.001), FCT-B (73.1±52.3 *vs*. 137.8±40.7 sec, *P* = 0.001). P300ERP (332.9±45.7 *vs*. 378.2±33.2msec, *P* = 0.001) and venous ammonia (67.1±45.7 *vs*. 103.3±37.2 *µ*mol/L, *P* = 0.001).

### Predictors associated with MHE

**Figure 1:**
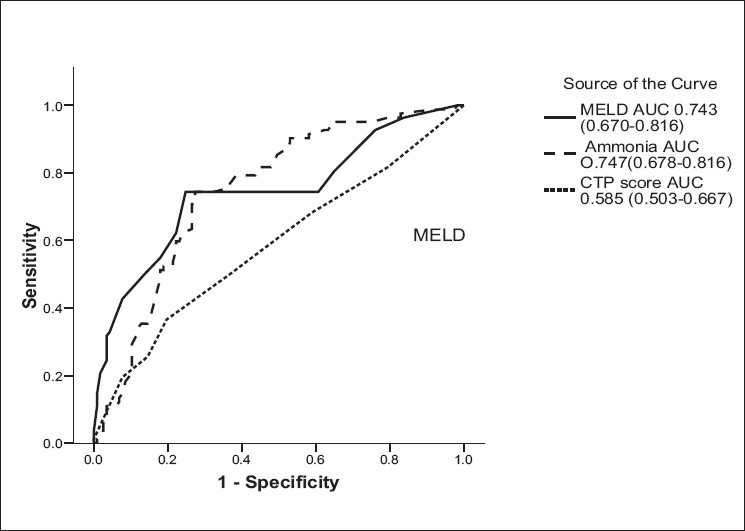
Receiver operating characteristic curve

MELD (17.9 ± 5.7 *vs*. 13.4 ± 4.2; *P* = 0.005), CTP score (8.4 ± 2.5 *vs*. 7.7 ± 2.2; *P* = 0.02), ammonia (104.8 ± 37.9 *vs*. 72.5 ± 45.2 *µ*mol/L; *P* = 0.001) and CFF (37.0 ± 2.8 *vs*. 41.0 ± 3.4 Hz; *P* = 0.001) were significantly higher in patients with MHE as compared to patients without MHE [Table 3]. Correlations of MHE with MELD, CTP score, CFF and venous ammonia level are shown in Table 4. Receiver operating characteristic (ROC) analysis was done to identify cutoff for MELD, CTP score and venous ammonia level [Table 5, Figure 1]. 91 patients (45.5%) had MELD > 15.5, 115 (57.5%) had CTP score > 7.5, while 93 (46.5%) had venous ammonia > 84.5 *µ*mol/L. On univariate analysis, MELD (8.52 [95% CI, 4.46-16.26; *P* = 0.001]), CFF (17.34 [95% CI, 8.16-36.85; *P* = 0.001]) and venous ammonia (7.80 [95% CI, 4.11-14.81; *P* = 0.003]) were associated with MHE; while CTP score (1.51 [95% CI, 0.85-2.69; *P* = 0.30]) was not found to be significant. On multivariate analysis MELD, CFF and venous ammonia were significantly associated with MHE [Table 6].

**Table 3:** Demographic, clinical and biochemical characteristics of patients with minimal hepatic encephalopathy and those with non-minimal hepatic encephalopathy

	MHE (*n*=82)	Non-MHE (*n*=118)	*P* value
Age (mean±S.D.) (years)	43.2 ± 11.4	42.2 ± 11.8	NS
Sex (M:F)	62:20	97:21	NS
P300ERP (ms)	392.3 ± 23.7	331.2 ± 23.6	0.001
Psychometric tests			
NCT-A (sec)	55.9 ± 8.1	29.9 ± 12.8	0.001
NCT-B	152.2 ± 20.9	65.3 ± 41.8	
FCT-A	74.6 ± 10.3	28.8 ± 13.8	
FCT-B	156.9 ± 15.2	56.4 ± 31.3	
CTP score	8.4 ± 2.5	7.7 ± 2.2	0.02
MELD score	17.9 ± 5.7	13.4 ± 4.2	0.001
CFF (Hz)	37.0 ± 2.8	41.0 ± 3.4	0.001
Ammonia (µmol/L)	104.8 ± 37.9	72.5 ± 45.2	0.001
Spontaneous shunts	19	13	NS

NCT: number connection test, FCT: figure connection test, CFF: critical flicker frequency, P300ERP: P300 event-related potential

**Table 4:** Correlation of minimal hepatic encephalopathy with Child-Turcotte-Pugh score, model for end-stage liver disease, venous ammonia and critical flicker frequency

Parameter	Spearman’s correlation coefficient	*P* value
CTP score	–0.143	0.044
MELD score	–0.411	0.001
Venous ammonia	–0.421	0.001
CFF	0.563	0.001

**Table 5:** Receiver operating curve for Child-Turcotte-Pugh score, model for end-stage liver disease, venous ammonia and critical flicker frequency for the diagnosis of minimal hepatic encephalopathy

Parameter	Cutoff	AUC (95% CI)	Sensitivity %	Specificity %	*P* value
CTP score	7.5	0.585 (0.503-0.667)	63.4	47	0.04
MELD score	15.5	0.743 (0.670-0.816)	74.4	75.2	0.001
Venous ammonia	84.5	0.747 (0.678-0.816)	74.4	72.6	0.001
CFF	39.0	0.830 (0.772-0.888)	72.9	88.6	0.001

**Table 6:** Univariate and multivariate analyses of predictors of minimal hepatic encephalopathy

Variables	Univariate		Multivariate	
	OR (95% CI)	*P* value	OR (95% CI)	*P* value
CTP score				
<7.5	1	0.306		
≥7.5	1.51 (0.85-2.69)			
MELD score				
<15.5	1	0.001	4.92 (2.29-10.55)	0.001
≥15.5	8.52 (4.46-16.26)			
Venous ammonia				
<84.5 µmol/L	1	0.003	2.49 (1.11-5.57)	0.001
≥84.5 µmol/L	7.80 (4.11-14.80)			
CFF				
≥39 Hz	1	0.001	8.83 (3.77-20.66)	0.001
<39 Hz	17.34 (8.16-36.85)			

## DISCUSSION

The prevalence of MHE in this study was 41%; and a MELD score >15.5, CFF <39 Hz and venous ammonia >84.5 *µ*mol/L are significantly associated with MHE.

MHE impairs patient’s daily functioning and quality of life. Patients with MHE have difficulties with attention, response inhibition and working memory, which are associated with driving impairment and high motor vehicle accident risk.[1–4] Controversy exists in literature regarding the correlation between severity of liver disease and prevalence of MHE. Many studies have shown correlation of impairment found in psychometric and neurophysiological tests with increasing severity of liver disease assessed by CTP class, while others find no such correlation.[7,9,23,29,32–34] In our study, 26/74 (35%) patients were in Child A grade; 26/72 (36%), in Child B grade; and 30/54 (56%), in Child C grade from among the total number of patients; so more patients with Child C grade had MHE as compared to the number of patients with Child A or B grade (*P* < 0.05). In a study by Yoo *et al*.,[23] there was no difference in the MELD scores between those with and without electroencephalogram (EEG) abnormalities (19.1 ± 6.9 *vs*. 15.4 ± 5.7; *P* = ns) or between those with normal and abnormal neuropsychometric examination results (17.6 ± 6.4 *vs*. 14.7 ± 5.7, *P* = ns); while the study by Meyer *et al*.[24] concluded that MELD scores were significantly and positively correlated with results of both trail-making tests (TMT-A and TMT-B) (r = 0.30, *P* < 0.001; and r = 0.18, *P* < 0.02, respectively), suggesting that increased disease severity is related to significantly slower performance. We also found MHE was significantly correlated with MELD scores (r = –0.411, *P* < 0.001). One of the important aspects of the MELD which is calculated from three biochemical variables (serum bilirubin, prothrombin time and creatinine) is that it has continuous variables and accounts for the spectrum of disease severity. However, using the most discriminative cutoff from the receiver operating characteristic curve may provide a model which might be a useful strategy in selecting candidates for any cirrhosis-related complication.[35–37] In our study, MELD score of 15.5 or above was found to be associated with MHE with a sensitivity of 74.4% and a specificity of 75.2% with ROC of 0.743 (95% CI, 0.670-0.816; *P* = 0.001). Although CTP score can be calculated easily, yet severity of ascites and HE is subject to individual variation. When we analyzed the CTP score association with MHE, cutoff of 7.5 showed a sensitivity of 63.4% and a specificity of 47% with ROC of 0.585 (95% CI, 0.503-0.667; *P* = 0.04), which was significantly lower than that for MELD. Although patients with higher MELD score (>15.5) have high probability of having MHE, yet we would like to emphasize that one should not exclude the possibility of MHE based solely on a low MELD score. Among our patients with a MELD score of ≤15, 21 (26%) had MHE.

CFF is a well-established neurophysiological technique that measures the ability of the central nervous system to detect flickering light, and which is directly influenced by cortical activity. In recent years, various studies have shown the utility of CFF for diagnosis of MHE.[27–29] In this study, at a cutoff of 39 Hz, specificity was 86.6% and sensitivity was 72.9%. Patients with CFF less than 39 Hz had significantly higher values of psychometric tests and P300ERP when compared with patients with CFF greater than 39 Hz. CFF was associated with MHE on both univariate and multivariate analyses.

Ammonia has been found to be a significant contributor to the pathogenesis of MHE in various studies. Ammonia and other neurotoxins act synergistically to induce a low-grade cerebral edema as a result of swelling of astrocytes, which is mainly due to increased intracellular content of glutamine, secondary to ammonia metabolism.[27] In this study also, venous ammonia was significantly higher in MHE patients as compared to non-MHE patients (104.8 ± 37.9 *vs*. 72.5 ± 45.2; *P* = 0.001), which correlates well with findings from other studies.[27,29] Venous ammonia was found to be correlated with MHE (r= –0.421, *P* = 0.001), and its sensitivity and specificity were 74.4% and 72.6%, respectively, with AUC of 0.747 (95% CI, 0.678-0.816; *P* = 0.001). It was found to be associated with MHE on multivariate analysis also.

We used abnormal psychometric test and P300ERP as a method of diagnosing MHE in the present study. Event-related P300 wave, which represents endogenous mechanism of stimulus processing, is the most consistent wave and can be considered the electrophysiological counterpart of the psychometric tests as both involve active use of the cognitive faculties. This method also excludes learning bias which is present in a follow-up study. Many studies favored event-related potential for the diagnosis of MHE in cirrhotics over psychometric test, whereas few studies showed no added benefit of P300 latency for the diagnosis of MHE.[38–43] P300ERP alone as a diagnostic method for MHE has limitations as it has been shown to be influenced by cirrhosis, significant changes in stimulus intensity and age. The use of a fixed cutoff will overrate the prevalence of alterations in old patients and underrate that in young patients.[41–46] So a battery of psychometric tests is recommended by Ferenci *et al*.[47] for the diagnosis of MHE.

To conclude, the prevalence of MHE in this study was 41%; and a MELD score > 15.5, CFF < 39 Hz and venous ammonia > 84.5 *µ*mol/L were significantly associated with MHE.
